# Follow-up of atheroma burden with sequential whole body contrast enhanced MR angiography: a feasibility study

**DOI:** 10.1007/s10554-016-0842-z

**Published:** 2016-01-25

**Authors:** Jonathan R. Weir-McCall, Richard D. White, Prasad G. Ramkumar, Stephen J. Gandy, Faisel Khan, Jill J. F. Belch, Allan D. Struthers, J. Graeme Houston

**Affiliations:** Division of Cardiovascular and Diabetes Medicine, Medical Research Institute, University of Dundee, Dundee, DD1 9SY UK; Department of Clinical Radiology, University Hospital of Wales, Cardiff, CF14 4XW UK; NHS Tayside Clinical Radiology, Ninewells Hospital, Dundee, DD1 9SY UK; NHS Tayside Medical Physics, Ninewells Hospital, Dundee, DD1 9SY UK

**Keywords:** Atherosclerosis, Peripheral arterial disease, Whole-body imaging, Magnetic resonance angiography, Disease progression

## Abstract

Assess the feasibility of whole body magnetic resonance angiography (WB-MRA) for monitoring global atheroma burden in a population with peripheral arterial disease (PAD). 50 consecutive patients with symptomatic PAD referred for clinically indicated MRA were recruited. Whole body MRA (WB-MRA) was performed at baseline, 6 months and 3 years. The vasculature was split into 31 anatomical arterial segments. Each segment was scored according to degree of luminal narrowing: 0 = normal, 1 = <50 %, 2 = 50–70 %, 3 = 71–99 %, 4 = vessel occlusion. The score from all assessable segments was summed, and then normalised to the number of assessable vessels. This normalised score was divided by four (the maximum vessel score) and multiplied by 100 to give a final standardised atheroma score (SAS) with a score of 0–100. Progression was assessed with repeat measure ANOVA. 36 patients were scanned at 0 and 6 months, with 26 patients scanned at the 3 years follow up. Only those who completed all three visits were included in the final analysis. Baseline atherosclerotic burden was high with a mean SAS of 15.7 ± 10.3. No significant progression was present at 6 months (mean SAS 16.4 ± 10.5, *p* = 0.67), however there was significant disease progression at 3 years (mean SAS 17.7 ± 11.5, *p* = 0.01). Those with atheroma progression at follow-up were less likely to be on statin therapy (79 vs 100 %, *p* = 0.04), and had significantly higher baseline SAS (17.6 ± 11.2 vs 10.7 ± 5.1, *p* = 0.043). Follow up of atheroma burden is possible with WB-MRA, which can successfully quantify and monitor atherosclerosis progression at 3 years follow-up.

## Introduction

Atherosclerosis is the pathological process underpinning the leading causes of morbidity and mortality in the western world. Despite the fact that it typically presents with symptoms localising to a single site, atherosclerosis is a systemic process, with those with disease presenting in one site at markedly increased risk for developing symptomatic disease at another site [1]. Peripheral arterial disease (PAD) is particularly prone to this with previous reports showing a high prevalence of silent myocardial infarctions, extra-site disease, and risk of future cardiovascular events [2–4].

Whole body magnetic resonance angiography (WB-MRA) has been proven to be useful for assessing the atheroma burden throughout the body in both healthy volunteers and patients with cardiovascular disease [5–7]. This has been shown to correlate well with the presence of obstructive coronary arterial disease, and as a predictor of future major adverse cardiovascular events [8–10]. However the ability to assess and quantify atheroma progression with WB-MRA has not been previously investigated. Therefore we set out to determine the feasibility of using WB-MRA to quantify and monitor atheroma progression in a population with PAD.

## Materials and methods

Institutional review board and research and development committee approval was granted for this study. All enrolled participants gave written informed consent. 50 consecutive patients referred for MRA of their lower limbs with PAD and symptomatic claudication were recruited. Disease severity was classified using the Fontaine scoring system. All patients underwent WB-MRA at baseline, with repeat WB-MRA scans being performed at 6 months, and 3 years after baseline. The baseline data of the full cohort have been described in detail previously, while the current article focuses on the longitudinal follow-up and assessment of the cohort [11].

### WB-MRA

At baseline and 6 months, all scanned patients underwent contrast-enhanced WB-MRA using a 1.5T MRI unit (Siemens Magnetom Avanto, Erlangen, Germany). Fifty consecutive patients were recruited to the study. Four patients were excluded due to inability to complete the MRI examination due to claustrophobia, leaving 46 participants who received the baseline scan. A total of 36 participants completed the 6 months follow-up study, and 26 returned for the 3 years follow-up scan. Drop outs were due to: two deaths; eight who had developed subsequent health complications and felt unable to continue; and ten who were uncontactable. At 3 years follow-up, 26 participants were scanned: n = 8 were scanned at 1.5T and n = 18 were scanned on a 3T MRI unit (Siemens Magnetom Trio, Erlangen, Germany).

Patients were imaged head first and supine in the magnet bore, with surface coils used to cover the entire body as follows: one head matrix; one neck matrix; one spine matrix; two body matrix; and one peripheral angiography. WB-MRA was performed using four separate stations for image acquisition, as below:

- Station 1—head, neck and thoracic vessels
- Station 2—abdominopelvic vessels
- Station 3—upper leg vessels
- Station 4—lower leg vessels

Full details of the technique are described in detail elsewhere [11], but in brief, the examination consisted of pre-contrast (‘mask’) and post-contrast MR imaging at all anatomical stations using a fast spoiled gradient echo (FLASH—Siemens, Germany) sequence. After the pre-contrast images for station 1 and 4 had been acquired, 20 ml of 0.5 M intravenous gadolinium based contrast agent (gadoterate meglumine, Guerbet, FR) followed by a 20 ml volume of normal saline was administered at 1 ml/s using an injector pump via a 20G venflon in the antecubital fossa using a dual injection method [12]. Following this, a fluoroscopic-guided post-contrast FLASH sequence was implemented at station 1 when the contrast agent reached the aortic arch. Immediately thereafter, the same FLASH acquisition was completed at station 4 on three consecutive occasions (to optimise the peak contrast enhancement at the lower limb vessels).

After a 10 min delay, the pre-contrast mask for stations 2 and 3 were acquired following which the second injection of contrast agent was delivered and flouroscopic guided triggering was used at the proximal abdominal aorta to define the start of the FLASH acquisition for station 2. Finally, FLASH data for station 3 were acquired immediately after completion of the station 2 sequence.

The resulting images were analysed by dividing the arterial tree into 31 distinct anatomical segments. Each segment was scored according to the maximal stenosis present at any point within it, using a 5 point scoring system:

- 0: Normal vessel
- 1: <50 % stenosis
- 2: 50–70 % stenosis
- 3: >70 % stenosis
- 4: Completely occluded vessel

Arterial segments which were not visualised with sufficient clarity for grading of the degree of stenosis were not analysed. To account for this, the final score was divided by the number of segments which had been successfully analysed (n), and then calculated as a percentage of the maximum possible score (see equation below) to produce a ‘standardised atheroma score’ (SAS) [13].

$$SAS = \left[ {\left( {\frac{\varSigma \,vessel\,scores}{n}} \right) \times \frac{1}{4}} \right] \times 100$$

**Fig. 1:**
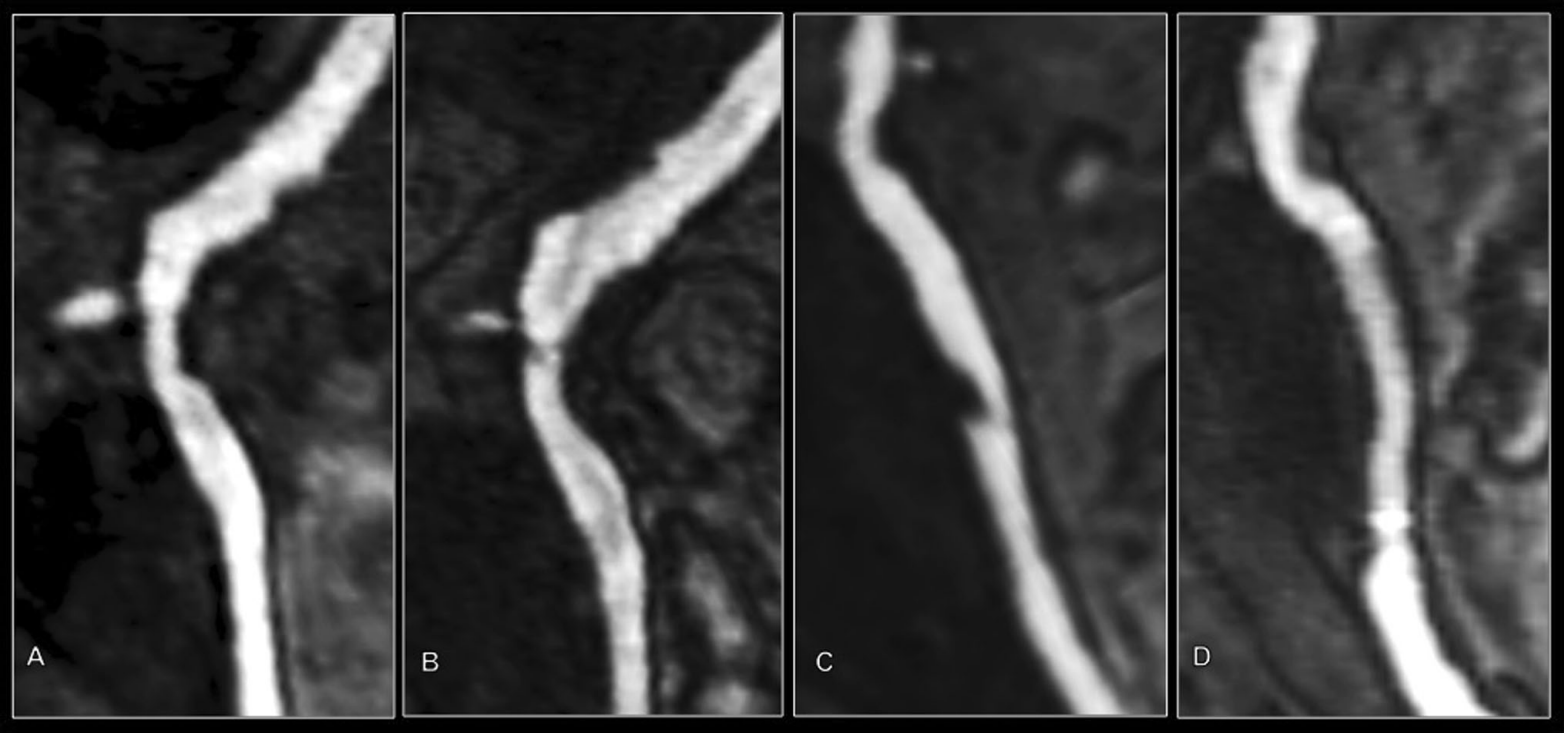
Examples of atheroma progression (**a,** **b**) and regression (**c**, **d**). MRA of the right iliac artery at baseline (**a**) showing a grade 2 (50–70 %) stenosis just distal to the internal iliac artery origin which has progressed to a grade 3 (70–99 %) stenosis at 3 years follow-up (**b**). MRA of the iliac artery in patient with interval angioplasty showing baseline grade 3 stenosis (**c**) which resolves on follow-up with only minor remaining luminal irregularity (grade 1) (**d**)

The 31 vessel segments were also subdivided into 5 anatomical territories: (1) the head and neck arteries; (2) the aorta; (3) the abdominal arteries; (4) the ilio-femoral arteries; and (5) popliteal and infrageniculate arteries (Fig. 1). Regional SASs were calculated for each anatomical territory.

WB-MRA analysis was performed using a diagnostic PACS radiological workstation (Carestream PACS Client Suite Version 10.1 sp1, Rochester, NY, USA) using the raw data in multiplanar reformat (MPR) analysis. Multiplanar visualisation was used in order to best visually estimate the degree of area stenosis. All images were analysed by a single radiologist with over 3 years experience in vascular radiology blinded to the patients’ clinical details. In order to determine repeatability of the scoring 26 scans from the final study visit were scored twice by this observer with a greater than 6 months interval between readings, and by two additional observers (one with over 3 years, and the other with over 20 years experience in vascular radiology). 2-way mixed, absolute agreement, average measure intraclass correlation co-efficient (ICC) for the whole body SAS was 0.94 (95 % CI 0.82–0.98) for intra-observer repeatability, and 0.89 (95 % CI 0.63–0.96) for inter-observer variability between the 3 observers (ICC > 0.75 = excellent, 0.4–0.75 = good, <0.4 = poor, <0.2 = slight).

### Ankle Brachial Pressure Index (ABPI) and carotid intima media thickness (CIMT)

These were performed as previously described [11]. Briefly, the CIMT was measured 1 cm proximal to the carotid bulb at a point where 1 cm of continuous intima media could be visualised. Both common carotids were measured twice and an average obtained.

ABPI was calculated using the highest of the two upper limb brachial blood pressures, and the higher of the two lower limb blood pressures.

### Statistical analysis

Categorical variables are presented as frequencies or percentages; normally distributed continuous variables are presented as means  ±  SD; and skewed variables are presented as median with interquartile range (IQR; 25–75th percentile). Normality of continuous variables was tested with the Shapiro–Wilk test. An independent sample *t* test was used to compare demographics between those with and without atheroma progression. Repeated measure ANOVA was used to analyse SAS progression between the baseline and 3 years follow up visit, with a post hoc analysis with bonferonni correction for differences between the three visits. A *p* value of <0.05 was considered significant. All statistical analyses were performed with IBM SPSS Statistics for Windows, version 21.0 (IBM Corp., NY, USA).

## Results

Fifty consecutive patients were recruited to the study. Four patients were excluded due to inability to complete the MRI examination due to claustrophobia, leaving 46 participants who received the baseline scan. A total of 36 participants completed the 6 months follow-up study, and 26 returned for the 3 years follow-up scan. Drop outs were due to: two deaths; eight who had developed subsequent health complications and felt unable to continue; ten who were uncontactable. Of the final 26, 73 % (n = 19) were male, with a mean age of 64.5 ± 9.5 years. A summary of demographic data is provided in Table 1. In terms of symptomatic PAD severity, 2 patients were Fontaine 1, 2 were Fontaine IIa, 35 were Fontaine IIb, 5 were Fontaine III and 2 were Fontaine IV. There was no significant differences in the demographics or baseline atheroma burden between those who completed all three visits and those who dropped out (*p* > 0.1 for all variables).

**Table 1:** Demographics and clinical characteristics of the study population

	Study population (n = 26)
Male (%)	19 (73 %)
Age (years)	64.5 ± 9.6
BMI (kg/m^2^)	28.4 (20.7–41.2)
Systolic BP (mmHg)	145 ± 11
Diastolic BP (mmHg)	82 (43–102)
Type 2 diabetes	5 (19 %)
Hypertension	18 (69 %)
Smoking status	
Current smoker	5 (19 %)
Ex-smoker	19 (73 %)
Non-smoker	2 (8 %)
Smoking pack years	38 ± 34
Medications	
Anti-hypertensive	19 (73 %)
Anti-platelet	22 (85 %)
Statin	22 (85 %)
Baseline SAS	15.7 ± 10.3
CIMT	0.94 ± 0.18
ABPI	0.82 (0.51–2.05)

Values expressed as mean ± SD, median (range) or N (%)

*BMI* body mass index, *BP* blood pressure, *SAS* standardised atheroma score, *CIMT* carotid intima media thickness, *ABPI* ankle-brachial pressure index

**Fig. 2:**
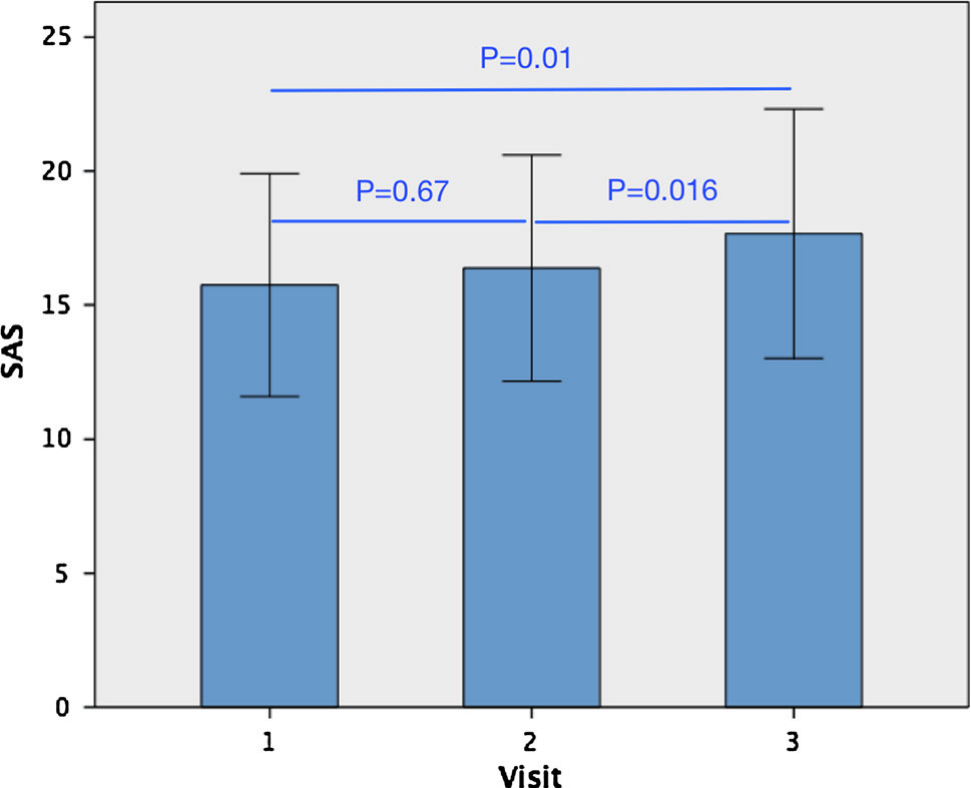
Comparison of atheroma score at baseline, 6 months and 3 years. *SAS* standardized atheroma score. *T-bars* represent 95 % confidence intervals

There were 2418 segments for analysis. Of the 2418 possible segments, 2387 (98.7 %) were considered to be of sufficient quality for analysis. At baseline SAS was 15.7 ± 10.3, with a mean SAS of 16.4 ± 10.5 at 6 months, and a mean SAS of 17.7 ± 11.5 at 3 years. No significant progression was present at 6 months with a change in SAS of 0.63 ± 0.51 (*p* = 0.67). This lack of significant progression persisted even when all 36 who underwent the first two scans were included in the analysis (mean progression 0.96 ± 1.08, *p* = 0.38). There was significant disease progression at 3 years with a mean progression in SAS of 1.92 ± 1.24 (*p* = 0.01). Over the 3 years period, this gave an annual progression rate in the atheroma burden of 4.1 % per year. Significant progression was also present between the 6 months SAS and the 3 years follow-up (mean progression 1.29 ± 0.84, *p* = 0.016)—see Fig. 2.

**Fig. 3:**
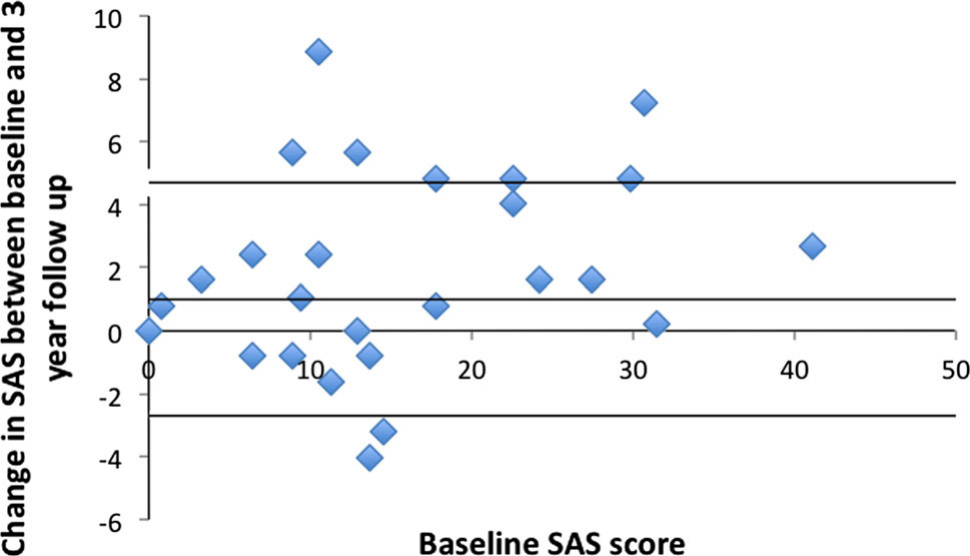
Bland Altman plot comparing baseline SAS (*x-axis*) with SAS change at 3 years (*y-axis*). The *mid*, *upper* and *lower line* represents the mean, and upper and lower 1.96*SD, of the change in SAS between baseline and 3 years follow up respectively

At 3 years eighteen participants showed progression in their atheroma score, while two were stable, and six showed an improvement (see Fig. 3). Out of the six whose atheroma score reduced, three had undergone one or more percutaneous interventions (one with femoral angioplasty, one with bilateral iliac angioplasties, one with bilateral femoral angioplasties with unilateral stent insertion). One patient had a single intervention between visit 1 and 2, one had a single intervention between visit 2 and 3, and one had 2 separate visits for interventional procedures with one between each visit. The remaining three had no intervention to explain their improvement. In comparison two participants demonstrated atheroma progression despite interval percutaneous intervention (one with two angioplasties and a stent inserted between the first two scans, the other with a common iliac stent inserted between the first and second scan). When the participants who were scanned at 1.5T on the final visit were compared with those scanned at 3T on the third visit there was no difference in the final visit atheroma burden (1.5T: 16.6 ± 9.2, 3T 18.1 ± 12.6, *p* = 0.75) or rate of progression (1.5T: 1.7 ± 2.7, 3T 2.2 ± 3.2, *p* = 0.71).

Whilst the whole body SAS demonstrated significant progression over the 3 years, when this was split into five distinct anatomical regions, no single region demonstrated significant progression on its own over the 3 years [(1) head and neck (mean difference 1.4 ± 4.7, *p* = 0.4); (2) aorta (mean difference 1.9 ± 4.3, *p* = 0.09); (3) the abdominal arteries (mean difference 0.96 ± 8.9, *p* = 01); (4) ilio-femoral arteries (mean difference 2.5 ± 5.1, *p* = 0.16); and (5) popliteal and infrageniculate arteries (mean difference 3.0 ± 3.0, *p* = 0.15), (see Table 2)].

**Table 2:** Change in whole body and regional SAS between baseline and 3 years follow-up

	Baseline	3 years	Mean difference	*p*
WB-SAS	15.7 ± 10.3	17.7 ± 11.5	2.0 ± 3.1	0.014
Head/neck-SAS	9.0 ± 11.2	10.4 ± 11.7	1.4 ± 4.7	0.4
Aorta-SAS	13.5 ± 8.5	15.4 ± 6.9	1.9 ± 4.3	0.09
Abdomen-SAS	9.2 ± 12.5	10.2 ± 11.7	0.96 ± 8.9	1
Ilio-femoral-SAS	32.2 ± 17.5	34.3 ± 19.7	2.5 ± 5.1	0.16
Run off-SAS	15.8 ± 19.6	18.8 ± 22.2	3.0 ± 3.0	0.15

Values expressed as mean (95 % CI)

*SAS* standardised atheroma score

Out of the baseline characteristics, statin therapy was significantly more common in the group showing no progression than those with progression (100 vs 79 %, *p* = 0.04). In addition, baseline SAS was significantly higher in those who progressed compared with those with stable disease/atheroma regression (17.6 ± 11.2 vs 10.7 ± 5.1, *p* = 0.043) (see Table 3).

**Table 3:** Comparison of baseline characteristics between those with and without atheroma progression at 3 years follow-up

	Progression	Stable/regression	*p*
N	18 (69 %)	8 (31 %)	
Male (%)	15(79 %)	4(57 %)	0.29
Age (years)	66.3 ± 10.3	59.6 ± 4.7	0.11
BMI (kg/m^2^)	28.7 (20.7–36.5)	28 (24.9–41.2)	0.41
Systolic BP (mmHg)	144.8 ± 11.4	148.6 ± 11.9	0.47
Diastolic BP (mmHg)	81 (43–102)	84 (68–95)	0.57
Pulse pressure	64.5 ± 14.3	65.9 ± 15.5	0.83
Hypertension	13 (68 %)	5 (71 %)	0.89
Type 2 diabetes	3(16 %)	2(29 %)	0.48
Current smoker	4 (21 %)	1 (14 %)	0.71
Ex-smoker	14 (74 %)	5 (71 %)	0.91
Non-smoker	1 (5 %)	1 (14 %)	0.46
Smoking pack years	30.4 ± 21.6	55.4 ± 50.1	0.1
Anti-hypertensive	13 (68 %)	6 (86 %)	0.35
Anti-platelet	17 (89 %)	5 (71 %)	0.39
Statin	15 (79 %)	7 (100 %)	**0.04**
Baseline SAS	17.6 ± 11.2	10.7 ± 5.1	**0.043**
CIMT	0.94 ± 0.19	0.93 ± 0.18	0.92
ABPI	0.8 (0.51–2.05)	0.84 (0.72–1.37)	0.85

The bold values indicate statistically significant (*p* < 0.05)

Values expressed as mean ± SD, median (range) or N (%)

*BMI* body mass index, *BP* blood pressure, *SAS* standardised atheroma score, *CIMT* carotid intima media thickness, *ABPI* ankle-brachial pressure index

## Discussion

In this feasibility study we have demonstrated the possibility of using WB-MRA to monitor and quantify atherosclerosis progression in a population with PAD. To the best of the authors knowledge, this is the first time that sequential WB-MRA has been used to quantify the progression rates of stenotic arterial disease. This adds to the current literature which shows that WB-MRA can detect significant arterial pathology outwith the lower limbs, more accurately quantifies stenotic disease in the lower limbs than ABPI, and can effect management changes in this population [3, 14, 15]. Additionally, the extra cost of extending standard lower limb MRA to include whole body imaging in combination with a cardiac MRI has been shown to be cost-effective due to a reduction in the requirement for down stream extra tests [16].

The ability to quantify and monitor atherosclerosis is desirable for several reasons. Previous studies have shown atheroma burden to correlate better with future cardiovascular events than traditional risk factors, and therefore holds potential as a useful prognostic marker. Furthermore, the ability to identify early changes in a small number of people holds potential to be a useful in early identification of drug efficacy. The benefits of identifying such markers is well evidenced in the rapid uptake in the use of cardiac MRI in the quantification of left ventricular mass and volumes in drug studies, where the high reproducibility results in a reduction in study size number of 55–93 %, with the observed changes in ventricular measures correlating well with outcomes [17, 18]. CIMT and carotid-femoral pulse wave velocity (cfPWV) have also been used as surrogate markers for assessment of atherosclerosis progression. Of these, CIMT is the best studied, with reported progression rates of 0.0147 mm/year [19]. However, due to the resolution of ultrasound being in the range of 0.1–0.3 mm, and a 5–10 % measurement error, individual progression monitoring is not possible and the number required for observational studies is still relatively high [20]. Progression rates of cfPWV have previously been reported as 8.1–14.7 mm/s/year observed in a population of 483 subjects over a 6 years period, thus also requiring large numbers and long time periods to demonstrate change [21]. The strength of the WB-MRA technique appears to arise in its systemic assessment. When the arterial system was subdivided into five clinically separate areas no significant change over the 3 years was apparent in any of them. It was only when all 31 arterial segments were combined that progression could be observed. This may in part be due to the small number of subjects in our current study meaning significant progression could not be observed in individual arteries or regions. While larger study numbers may allow regional assessment of progression, this detracts from the two strengths of the whole body MRA technique, the first being its systemic assessment of a systemic disease process, and the second being its potential to reduce study sizes by detecting changes in a smaller number of individuals. While whole body CTA has previously been used to derive a similar whole body atheroma score, and has the benefit of providing coronary artery assessment at the same time, it is associated with a high radiation dose and a relatively high contrast dose [22].

The small number of patients who completed the protocol limits the generalisability of the results. However the current study was to determine feasibility rather than to definitively quantify atheroma progression rates. Despite the small number of participants, significant disease progression could still be observed. The validity of our findings is strengthened by the observation that those with atheroma progression were significantly less likely to be prescribed statins and to have higher SAS on their baseline WB-MRA. This is entirely consistent with studies in coronary artery calcium (CAC) scoring—a measure of calcified atheroma burden within the coronaries—showing higher CAC progression in those with higher baseline CAC [23], and lower progression rates in those on statins [24]. While a high drop out rate is problematic and raises concerns about patient acceptance of the technique, a previous study has shown higher acceptance of WB-MRA over digital subtraction angiography and thus the high attrition rate may be due to the high morbidity of the patient population studied, indeed half of our observed attrition rate was due to declining health [25].

Larger studies will be required to verify the reproducibility of these results, and also to explore the applicability in other cardiovascular disease cohorts, especially as PAD is known to have a higher atheroma burden, and therefore is likely to have a higher and therefore more easily measurable progression in atheroma burden. Additionally, the clinical significance of the WB-MRA progression data observed in this study needs to be established.

The current study has several limitations. The population is heterogenous as all clinical referrals for claudication assessment were recruited rather than a specifically defined disease severity. Despite this the majority of the participants were Fontaine IIb, and this heterogeneity also better reflects the spectrum of disease seen in routine clinical practice. In addition our analysis comparing the progression and regression groups could be confounded by the inclusion of participants who underwent interventional procedures, as this group could be considered clinical progressors due to their need for intervention, while those with no intervention and regression would be considered as true (or at least medically induced) regressors. However our results are commensurate with prior work in CAC [23, 24], and the study was neither designed nor of sufficient size to undertake this type of subgroup analysis. Further work is thus required to extricate the true effects of medical intervention. The magnetic field strength used for baseline and 3 years follow-up examinations was different in some cases for operational reasons. All participants were scanned using the 1.5T machine at baseline and 6 months, and 70 % of participants were scanned at 3T at the final 3 years follow-up. While this could potentially account for some of the temporal changes observed, previous studies have reported no significant differences in stenosis assessment between the two field strengths [26]. In addition there were no differences in progression rates between those scanned at 1.5T at all visits and those scanned at 3T at follow-up.

Whole body magnetic resonance angiography is a lumenographic technique, thus early wall thickening and extra-luminal plaque formation will be missed potentially underestimating disease. Whilst techniques such as isotropic black blood imaging may hold the potential for analysis of early wall thickening and stenosis assessment, this technique has yet to be used and validated in whole body vascular assessment, unlike the current angiographic technique. Furthermore other scores such as the Dukes score and modified Dukes score for invasive coronary angiography and CT coronary angiography have well demonstrated the strength of lumenographic techniques for quantification of disease burden and future prognosis [27].

## Conclusion

Whole body contrast-enhanced magnetic resonance angiography allows monitoring and quantitative assessment of global plaque burden over time. In patients with PAD, significant increases in atheroma burden can be observed over a 3 years period.
